# Supplementary figures and images for: The role of aquaporin-4 in optic nerve head astrocytes in experimental glaucoma
Source: PLoS One. 2021 Feb 2;16(2):e0244123. doi: 10.1371/journal.pone.0244123 (PMC7853498; doi:10.1371/journal.pone.0244123)

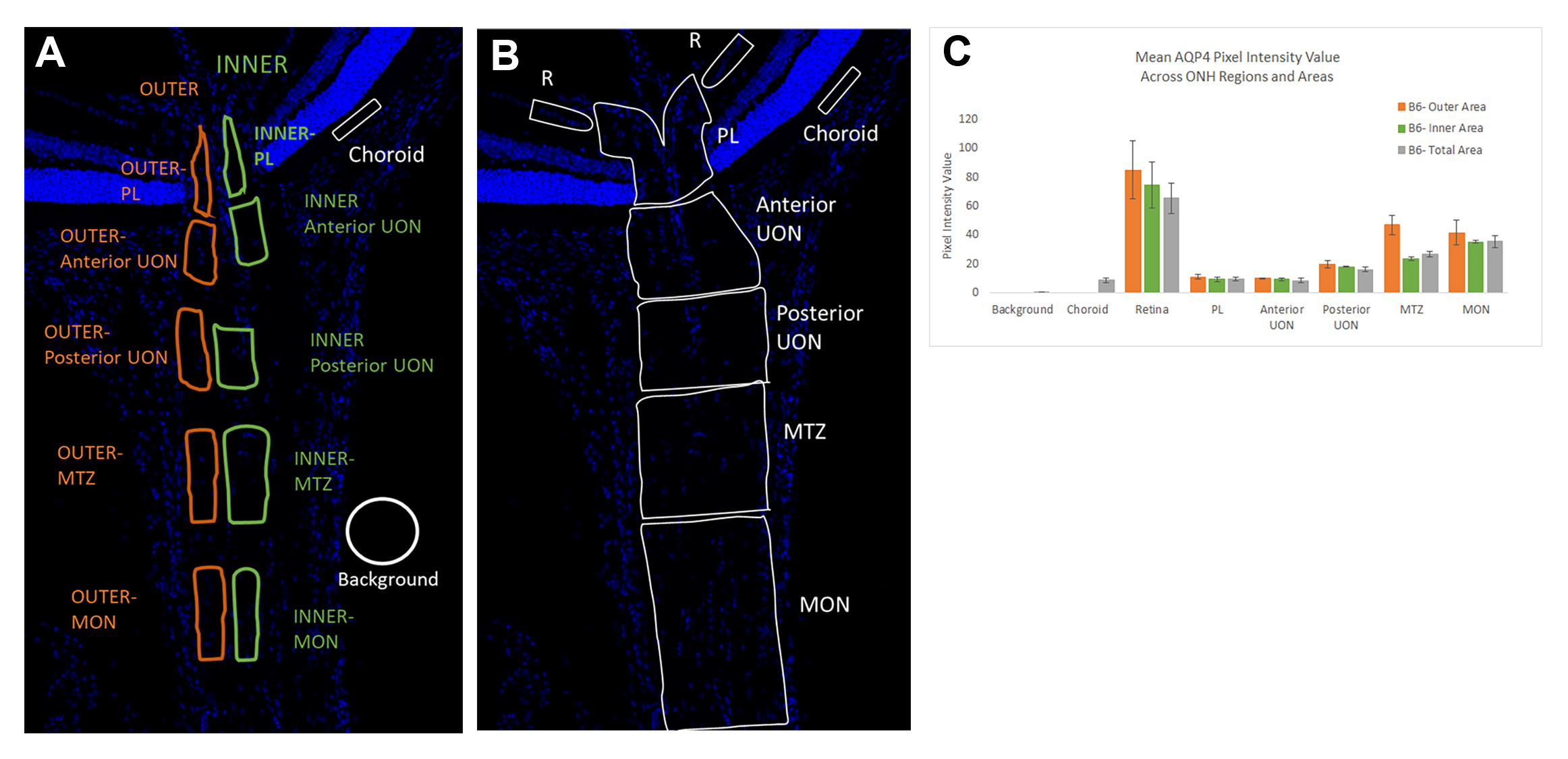

Supplement: S1 Fig — Schematic shows cryopreserved C57BL/6 control (B6) optic nerve head tissue stained with DAPI (blue) with outlined regions chosen for pixel intensity brightness analysis. First schematic (A) was divided into outer (orange) and inner (green) regions at the pre-lamina (PL, from vitreoretinal surface to a line joining the two endpoints of BMO), anterior unmyelinated optic nerve (anterior UON, from BMO to 100 μm posteriorly), posterior unmyelinated optic nerve (posterior UON, from 100 μm to 200 μm posteriorly), myelin transition zone (MTZ, from 200 μm to 350 μm posteriorly), and the myelinated optic nerve (MON, from 350 μm to the end of the section). Background area (white circle, A) and choroid (white rectangle, B) were used as AQP4 negative controls. Second schematic (B) shows the total area (gray outlines) of the regions; retina, choroid, PL, anterior-UON, posterior-UON, MTZ and MON. (C) Mean AQP4 pixel intensity value (PIV) graph plots 8 regions calculated using FIJI software in B6 control nerves immunostained for AQP4 in the three areas; outer area (orange bars), inner area (green bars) and total area (gray bars) as defined in (A) and (B). Standard error bars are plotted. Gray dotted line identifies the AQP4 background level in choroid. Scale Bar: 100 μm (A,B). (TIF) [file pone.0244123.s001.tif]

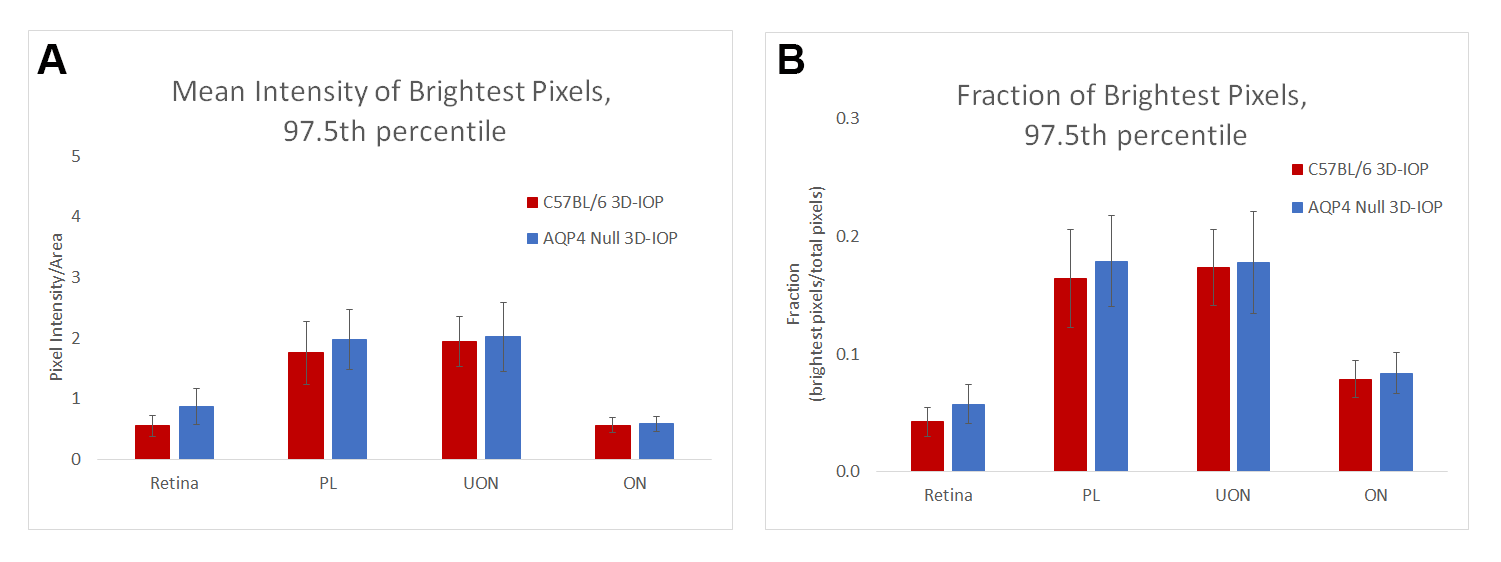

Supplement: S2 Fig — Amyloid precursor protein (APP) labeling assessed axonal transport block at 3D-IOP in four regions of the mouse optic nerve head: retina, pre-lamina (PL), unmyelinated optic nerve (UON) and myelinated optic nerve (MON). B6 (red) and AQP4 null (blue) nerves had similar transport block at PL and UON in the two key metrics, mean intensity of brightest pixels at the 97.5th percentile (A) and fraction of brightest pixels at the 97.5% percentile (B). (TIF) [file pone.0244123.s002.tif]
