# Supplementary material for: The role of aquaporin-4 in optic nerve head astrocytes in experimental glaucoma
Source: PLoS One. 2021 Feb 2;16(2):e0244123. doi: 10.1371/journal.pone.0244123 (PMC7853498; doi:10.1371/journal.pone.0244123)
Supplement: Attachment — Submitted filename: AQP4 Response to Reviewers_1.6.2021.docx [file pone.0244123.s003.docx]

**Response to Reviewers for Manuscript:** The Role of Aquaporin-4 in Optic Nerve Head Astrocytes in Experimental Glaucoma

**Corresponding Author:** *Elizabeth Kimball*

**Date:** 1/6/2021

**Comments to the Author**

1. Is the manuscript technically sound, and do the data support the conclusions?

The manuscript must describe a technically sound piece of scientific research with data that supports the conclusions. Experiments must have been conducted rigorously, with appropriate controls, replication, and sample sizes. The conclusions must be drawn appropriately based on the data presented.

Reviewer #1: Yes

Reviewer #2: Yes

2. Has the statistical analysis been performed appropriately and rigorously?

Reviewer #1: Yes

Reviewer #2: Yes

3. Have the authors made all data underlying the findings in their manuscript fully available?

The [PLOS Data policy](https://nam02.safelinks.protection.outlook.com/?url=http%3A%2F%2Fwww.plosone.org%2Fstatic%2Fpolicies.action%23sharing&data=04%7C01%7Cfcone1%40jhmi.edu%7C5ebcf4fa2c614013ae5308d8abfd118c%7C9fa4f438b1e6473b803f86f8aedf0dec%7C0%7C0%7C637448451312446476%7CUnknown%7CTWFpbGZsb3d8eyJWIjoiMC4wLjAwMDAiLCJQIjoiV2luMzIiLCJBTiI6Ik1haWwiLCJXVCI6Mn0%3D%7C1000&sdata=zZxlkvAF%2FRUcuCukHeGmHeohBNziNUeA7zYzN%2Fvm8EI%3D&reserved=0) requires authors to make all data underlying the findings described in their manuscript fully available without restriction, with rare exception (please refer to the Data Availability Statement in the manuscript PDF file). The data should be provided as part of the manuscript or its supporting information, or deposited to a public repository. For example, in addition to summary statistics, the data points behind means, medians and variance measures should be available. If there are restrictions on publicly sharing data—e.g. participant privacy or use of data from a third party—those must be specified.

Reviewer #1: Yes

Reviewer #2: Yes

4. Is the manuscript presented in an intelligible fashion and written in standard English?

PLOS ONE does not copyedit accepted manuscripts, so the language in submitted articles must be clear, correct, and unambiguous. Any typographical or grammatical errors should be corrected at revision, so please note any specific errors here.

Reviewer #1: Yes

Reviewer #2: No

5. Review Comments to the Author

Please use the space provided to explain your answers to the questions above. You may also include additional comments for the author, including concerns about dual publication, research ethics, or publication ethics. (Please upload your review as an attachment if it exceeds 20,000 characters)

Reviewer #1: The manuscript demonstrates that aquaporin does not have beneficial nor detrimental effects in a mouse model of glaucoma. This information provides insight into the distribution of aquaporin in the retina, optic nerve disk and optic nerve. The authors use an animal mouse model of glaucoma by inducing elevated intraocular pressure via bead occlusion. This glaucoma model was implemented in C57BL6 controls and AQP4 null mice. The authors used fluorescent immunohistochemistry to analyze the distribution of aquaporins in the eye and optic nerve and quantitative real-time PCR for measuring mRNA expression of aquaporins. Axonal transport was assessed through the movement of amyloid precursor protein.

Consider revising this sentence in introduction. Three AQP types have been described in astrocytes; AQP1, AQP4 and AQP9, aquaglyceroporin.

- *This sentences has been corrected and now reads: “Three AQP* ***types are known to express*** *in astrocytes ; AQP1, AQP4, and AQP9* ***(also referred to as, aquaglyceroporin which is permeable to glycerol and other small uncharged solutes).***

Include a brief overview of how the bead injections were carried out including vehicle used and where the microbeads were procured from (company).

- *The paragraph now reads: “One anterior chamber was injected with Polybead Microspheres (Polysciences, Inc., Warrington, PA, USA),* ***suspended in sterile PBS (phosphate buffer saline solution),*** *consisting of 2 µL of 6 µm diameter beads, then 2 µL of 1 µm diameter beads, followed by 1 µL of viscoelastic compound (10 mg/ml sodium hyaluronate, Healon; Advanced Medical Optics Inc., Santa Ana, CA). Injections were made with a 50 µm tip diameter glass cannula, connected to a Hamilton syringe (Hamilton, Inc., Reno, NV). The glass cannula was kept in place for 2 minutes to prevent the egress of beads after withdrawal. The contralateral eye was used as control. No animal was excluded from our study once the bead injection was completed.”*

Specify what type of knock-out was used (constitutive, conditional)?

- *The AQP4 null animals studied here are now referred to as “Aquaporin 4* ***constitutive knockout*** *(AQP4 null)”.*

*Animal numbers have been corrected for the gene expression portion of this manuscript (previously it stated 6 animals were studied, however, the correct total is* ***10****). Both the total animal count, and Table 1 (Mouse Groups) have been amended.*

Reviewer #2: Although this paper contains some important information on aquaporin channel expression in a mouse model of glaucoma, it requires minor revision. The manuscript should be revised with proper English grammar.

- *The manuscript has been revised for grammatical errors.*

*Two manuscript versions have now been uploaded- a “Tracked Changes” copy and an accepted changes copy.*

*Thank you,*

*Elizabeth Kimball*
